# The *trans*-influence in gold chemistry from a catalytic perspective

**DOI:** 10.3762/bjoc.22.66

**Published:** 2026-06-01

**Authors:** Manfred Bochmann

**Affiliations:** 1 School of Chemistry, University of East Anglia, Norwich Research Park, NR4 7TJ, Norwich, United Kingdom

**Keywords:** catalysis, gold, ligands, reaction mechanism, *trans*-influence

## Abstract

Gold catalysis has developed into an increasingly important method in organic synthesis. Especially the potential of gold(I,III) redox systems and gold(III) complexes has more recently become a focus of interest. Although often underestimated or not considered, the ligand *trans*-influence is a crucial factor in determining the reactivity of gold(III) compounds; it modifies not only bond polarisation, dissociation energies and thermal stability but also controls the nature of the gold frontier orbitals. This Perspective highlights recent insights into the origins and consequences of the ligand *trans*-influence and its importance for the energetics of gold-catalysed reactions, with special emphasis on the *trans*-influence in gold(III) hydrides and recent mechanistic insights.

## Introduction

Gold catalysts have emerged as catalysts of choice in a multitude of organic transformations, not least due to their high functional group tolerance. Their applications have been widely documented in monographs and special themed journal issues, see for example [1–10]. Of special interest here are gold complexes supported by bidentate and tridentate ligands; their chemistry has been the subject of a number of reviews, covering different aspects of structure, bonding and reactivity in catalytic reactions [11–19]. There is however one important aspect of gold chemistry that is often under-appreciated: the strong control of reactions by the influence of a given ligand on another ligand in *trans* position, known as the *trans*-influence. This effect is probably more dominant in gold chemistry than in most other transition-metal complexes; it can on occasions appear even more important than the formal oxidation state and d-electron count. Tilset and co-workers have recently summarised the importance of the *trans*-influence in C^N complexes and their role in catalytic applications [20].

While not aiming to be comprehensive, this Perspective seeks to highlight pertinent examples demonstrating the importance of the ligand *trans*-influence in gold chemistry, with special emphasis on the reactivity of gold(III), and its influence on gold-mediated reaction mechanisms. Following a brief discussion of general considerations, this Perspective examines how ligand structure and the resulting *trans*-influence determine reactivity and stability of gold(III) complexes supported by tridentate (pincer) ligands and in bidentate chelates, including electronic aspects such as photoluminescence and the spectroscopic and reactivity properties of gold(III) hydrides, before moving on to *trans*-influence enabled insertion reactions and catalytic applications.

## Perspective

### General considerations

“*Trans-influence*” is a term applied to the weakening or strengthening of a metal–ligand bond M–X as the result of another ligand L in *trans* position to X. The term refers to a ground-state property of a complex, such as M–X bond dissociation energy and bond length, and is therefore dictated by thermodynamics. “*Trans*-influence” is closely related to but distinct from the “*trans-*effect”, which describes the effect of a ligand L on the rate of substitution of another ligand in *trans* position, most prominently found in square-planar d^8^-complexes, notably those of platinum(II) [21]. This is a kinetic parameter and therefore combines ground-state energetics with the structure and bonding in the transition state. As summarised in an early review by Appleton, Clark and Manzer [22], both σ-donation and π-effects of the ligands are important.

Ligands with strong *trans*-influence are typically either strong π-acceptors such as CO and CN^−^, and strong σ-donors, like trialkylphosphines, anionic carbon and hydride ligands. N- and O-lone pair donors (pyridine, water) are weak ligands. The following indicative series is typical for Pt(II), although there may be slight variations in the specific ordering depending on the complex type under investigation:

CO, CN^−^, C_2_H_4_ > P(alkyl)_3_ > PPh_3_ > H^−^ > CH_3_^−^ > C_6_H_5_^−^, NO_2_^−^, I^−^ > Br^−^, Cl^−^ > py, NH_3_, H_2_O

Catalyst precursors in gold chemistry most commonly consist either of linear, two-coordinate Au(I) complexes LAuX, or square-planar Au(III) complexes, often supported by bidentate or tridentate ligands. In both cases the *trans*-influence plays an important role, affecting reaction rates, thermal stability, reaction pathways and selectivity.

Various approaches have been taken to quantify these effects. Given the ubiquitous nature of gold(I) complexes LAuX and the fact that in such systems steric effects tend to be negligible, many studies naturally focused on the assessment of the ligand influence in linear compounds. For example, in 1977 Jones and Williams correlated the ^35^Cl nuclear quadrupole resonance of LAuCl complexes with the electronic properties of L, for a series of alkyl and arylphosphines, phosphites, isocyanides, N-donors and Cl^−^ [23]. The results suggested that σ-bonding effects were dominant, although π-bonding also played a role. Interestingly, the results differed substantially from similar studies on platinum(II) complexes, partly because the Pt(II) d^8^ system enables a greater π-contribution, and partly because in square-planar geometries there is always also the influence of two *cis*-ligands. Melpolder and Burmeister used solution infrared spectroscopy to determine the ratio of N-bonded and S-bonded thiocyanate ligands in LAuSCN/NCS as a function of L. As expected, ligands L with a strong *trans*-influence, such as electron-rich trialkylphosphines, increased the proportion of N-bonded thiocyanate [24].

More recently, Espinet and co-workers used solution microcalorimetry with in-situ generated HI to provide bond dissociation energy scales for the Au–C bonds in R–Au(PPh_3_) (R = Me, aryl or alkynyl), and further to determine the effect of ligands L in the protolysis of LAuPh, where reaction enthalpies were shown to decrease in the order P(OPh)_3_ > PPh_3_ ≈ PMe_3_ > IPr > PCy_3_ (IPr = 1,3-bis(2,6-diisopropylphenyl)imidazol-2-ylidene). Depending on L these protolysis enthalpies with HI span a range of 50 kJ/mol. The Au–C values correlate well with the corresponding C–H bond dissociation energies [25], not unexpectedly perhaps given the similarity of electronegativities (Au, 2.54; C, 2.55; H, 2.20).

**Scheme 1:**
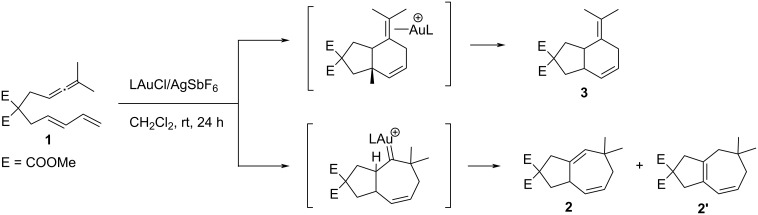
Assessment of ligand influence by kinetic competition experiments.

Toste et al. selected the ratio of [4 + 3] vs [4 + 2] alkene–allene cycloadditions as a probe for ligand effects, employing a range of phosphine ligands in the LAuCl/AgSbF_6_ system (Scheme 1). Cyclisation of the diene–allene substrate **1** gives a mixture of **2**, **2'** and **3**. Depending on the phosphine or phosphite employed, the product ratio **3**/**2** + **2'** was found to vary from 9.5:1 to 1:9. These ratios result in a series of ΔΔ*G*^‡^ values which linearly correlate with the calculated Au–Cl distances. These distances are in the main subject to electronic effects by L, perturbed by subtle steric interactions with the bulky phosphines. The same interactions are likely to operate in the rate-determining steps during the formation of **2** and **3**. This correlation proved to be of predictive value in the optimisation of product selectivity with as yet untested ligands [26].

Using kinetic competition experiments is a particularly sensitive method for assessing ligand effects in catalysis, since they are able to quantitatively reflect even small energy differences that arise from a combination of steric and electronic factors. Equally important is the use of calculated rather than crystallographically determined bond-length parameters to establish a scale of ligand characteristics. As Budzelaar and co-workers have pointed out [27], crystallographically determined bond distances, for example Au–N distances, which are quite sensitive to *trans*-influences but are rather “soft”, are subject to unsystematic crystal packing, conformational and substituent effects which are of a similar order of magnitude to the *trans*-influence that the correlation is trying to determine. Caution is therefore required in interpreting crystallographic bond lengths, and it is important to eliminate such factors by resorting to calculated bond distance data.

### Ligand structures and properties

**Scheme 2:**
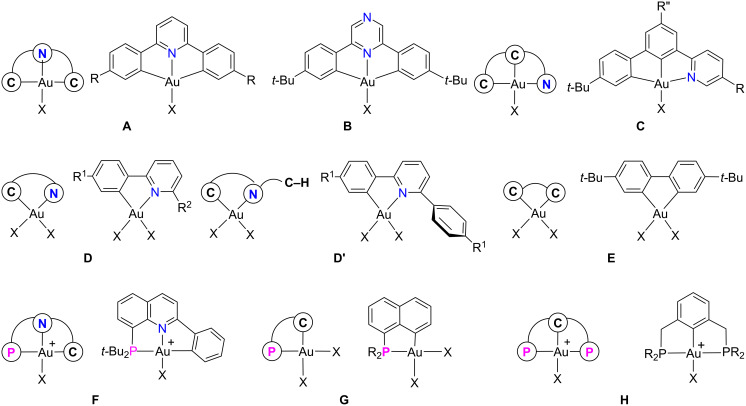
Ligand types employed in the stabilisation of gold(III) complexes.

For the exploration of the reactivity and catalytic applications of gold(III) compounds the use of tridentate (pincer) and bidentate ligands has proved particularly successful. A number of pertinent structural types **A**–**H** and the symbolic representations used in this Perspective are shown in Scheme 2. The choice of the donor *trans* to the reactive ligands X is crucial; for example, while C^N^C and C^C^N ligand frameworks look like simple structural isomers, their reactivity and analysis of their bonding has shown that replacing the weak N(pyridine) donor by strongly donating anionic carbon leads to profound changes in gold orbital ordering, often with drastic consequences for reactivity and thermal stability.

**Scheme 3:**
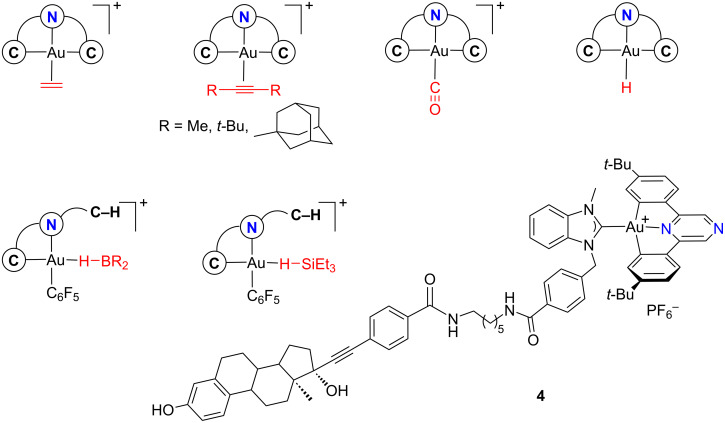
Au(III) π- and σ-complexes stabilised by C^N^C and C^N chelate ligands [28–32] and an example of a gold(III) complex targeting oestrogen-receptor positive (ER+) breast cancer cells [11,33–36].

Tridentate C^N^C ligands have been particularly successful in allowing the isolation of species that have often been postulated in catalytic cycles but have remained elusive until recently. These include the first gold(III) ethylene complex, some 180 years after its platinum(II) congener, K[PtCl_3_(ethylene)] [28], as well as the first examples of gold(III) alkyne [29], CO [30], hydride [31] and σ-complexes [32] (Scheme 3).

The olefin complexes were isolated as microcrystalline powders which as dry solids are fairly stable in air at room temperature. The olefin bonding is mostly via π-donation. There is minimal back-bonding, as indicated by the low barrier of rotation of the ethylene complex, which is about 10 times smaller than that for a comparable platinum(II) ethylene complex [12]. [(C^N^C)Au–CO]^+^ is hitherto the sole example of a CO complex of gold(III); it was isolated as a microcrystalline yellow solid that decomposes >−10 °C. The CO stretching frequency of 2167 cm^−1^ is 24 cm^−1^ higher than that of free ^12^CO and substantially higher than that of [(C^N^N)Pt–CO]^+^ (2094 cm^−1^). The calculated Au–CO bond enthalpy of −45.5 kcal/mol is about 7 kcal/mol less than that of Pt–CO. Bonding analysis of these CO complexes indicates mainly σ-donation but little back-donation, with donation/back-donation (*d/b*) ratios much larger than in the comparable Pt(II) complex [(C^N^N)Pt–CO]^+^. DFT studies found no evidence for π-bonding contributions to the Au–CO bond in the highest occupied molecular orbitals (HOMO, HOMO−1, HOMO−2, HOMO−3). According to natural bond orbital analysis, the Au–CO coordination involves a single bond, with major contributions from the gold 6s, 6p_y_, and 6d_x²−y²_ molecular orbitals [12,30]. The Au–CO adduct is therefore best regarded as a σ-complex. These differences are crucial for reactivity; for example, while the Pt–CO complex could be recrystallised from boiling methanol, the Au–CO complex is extremely sensitive to nucleophilic attack by water or alcohols (e.g., water–gas-shift, vide infra).

The high Lewis acidity of a cationic gold(III) centre must not be underestimated. For example, cleavage of one of the Au–phenyl bonds in **A** (Scheme 2) with a strong acid provides access to the gold borane and silane σ-complexes [(C^N–CH)Au(C_6_F_5_)(H–X)]^+^, X = B(O_2_C_2_Me_4_) or SiEt_3_ (Scheme 3) [32]. In the presence of traces of moisture, the Au(III)^+^ Lewis acidity may well give rise to proton-catalysed reactions, which can masquerade as gold catalysis.

On the other end of the stability scale are molecules such as complex **4**, an oestradiol-decorated metal complex conjugate, where the weak *trans*-ligand pyrazine strengthens the gold–carbene bond. Compound **4** shows increased take-up into oestrogen-receptor positive (ER+) breast cancer cells for improved anticancer activity [33]. Similar chelate and pincer gold(III) complexes are potential anticancer therapy targets [34–36].

**Scheme 4:**
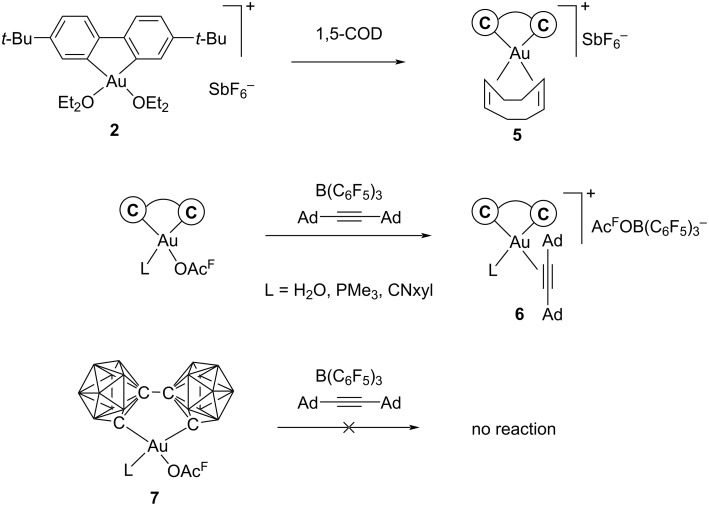
Gold(III) C^C chelate complexes.

In their assessment of the *trans*-influence of the C^N^C pincer in comparison with the C^C biphenylyl dianion Chambrier et al. have demonstrated that apart from the donor strength the electron-withdrawing character and the π-acceptor capacity of the chelate ligand can play an important role [37]. This was found to be the reason why [(C^C)Au(diene)]^+^ complexes (diene = 1,5-cyclooctadiene (**5**) or norbornadiene) are thermally stable and could be crystallographically characterised, while [Me_2_Au(1,5-COD)]^+^ decomposes >0 °C [38]. The total binding enthalpies in the [(C^C)AuL_2_]^+^ fragments closely resemble those of Me_2_PtL_2_, and are significantly lower than those of the Me_2_Au^+^ analogues (L = OH_2_, OMe_2_, HC≡CH, MeC≡CMe; L_2_ = 1,5-COD, NBD). Compared to [(C^N^C)AuL]^+^, the average bond enthalpies *trans* to C^(−)^ per L in [(C^C)AuL_2_]^+^ are only about 40–60% of the values *trans* to N in the C^N^C system. Simple n-donors like ethers are less affected, while for CO as a strong π-acceptor the bond enthalpy decreases to about 35%. Steric factors also come into play; together these effects explain why stable mono-alkyne complexes [(C^C)Au(alkyne)L]^+^ **6** could be isolated but bis(alkyne) complexes are inaccessible (Scheme 4). Similarly, the formation of the dicarbonyl complex [(C^C)Au(CO)_2_]^+^ would be endothermic. Extending the range of dianionic C^C chelates to dicarboranyls highlights the effects of electronic characteristics. For example, while the removal of a trifluoroacetate ligand by B(C_6_F_5_)_3_ can usually be relied upon to quantitatively create a vacant coordination site [27–29], in the case of **7** this method unexpectedly fails, according to bond energy calculations due to the electron-accepting fluoroaryl-like character of the dicarboranyl ligand [27].

### Ligand-dependent luminescence properties

**Figure 1:**
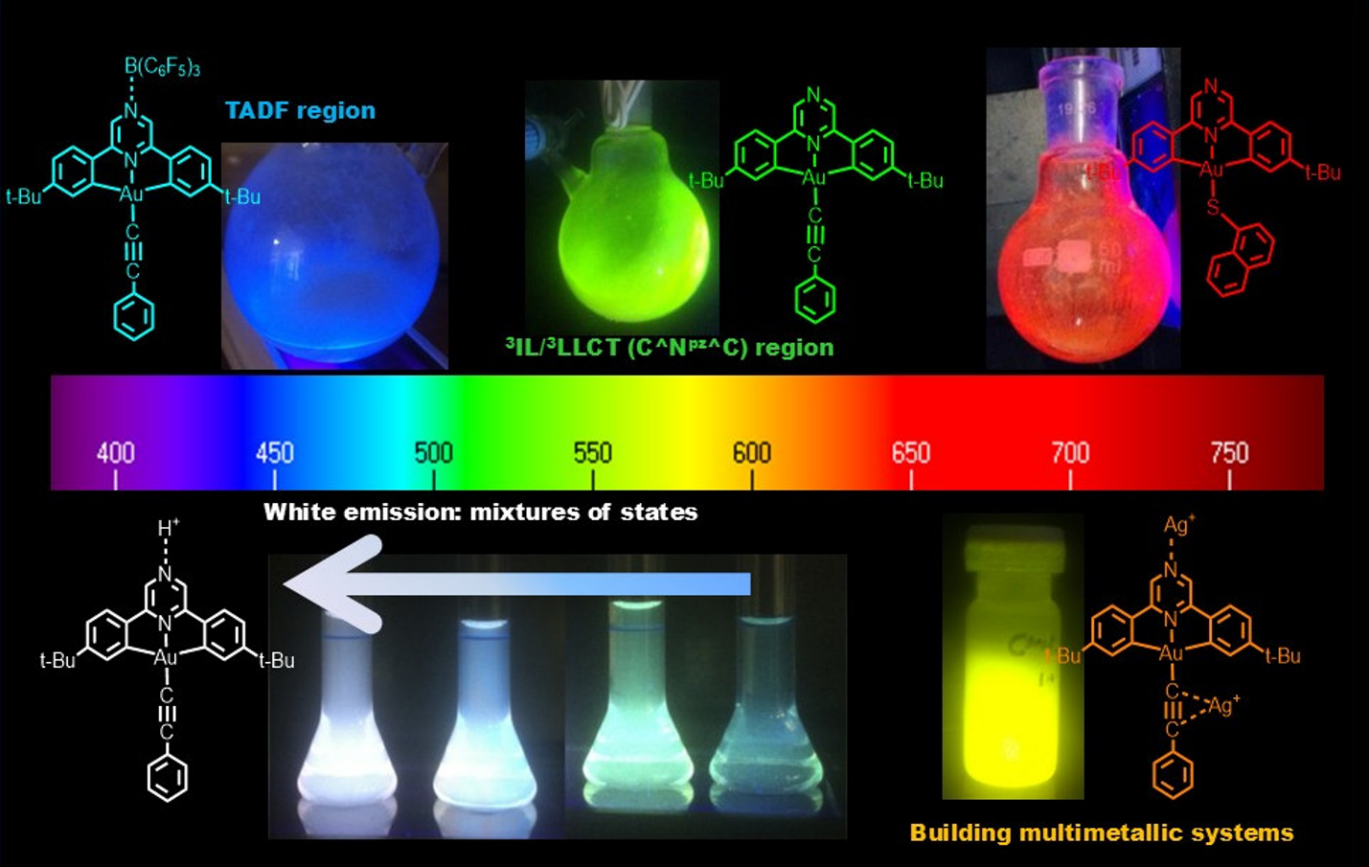
Examples of photoemissive gold(III) pyrazine complexes. Dr. J. Fernandez-Cestau is gratefully acknowledged for assistance with this figure.

The pincer ligands described above are of course themselves subject to the influence of *trans* ligands. They also differ in other respects, notably in their π-acceptor capacity. This leads to interesting photoluminescence effects. For example, while (C^N^py^^C)AuCl shows poor photoemissions in the solid state, this changes if Cl is replaced by strong donors, notably aryl, alkynyl or thiolate anions [39–40]. These raise the metal d-levels, thereby reducing their capacity to provide non-radiative decay pathways, and enable photoexcitation by ligand-to-ligand charge transfer (LLCT). The more electron-rich (C^C^N)AuCl is itself photoemissive and produces phosphorescence due to intraligand (^3^IL) excitation [41–43]. Gold C^C^C(carbene) alkynyl complexes are similarly strongly emissive [43]. Since the π–π* gap of pyrazine is 0.95 eV smaller than that of pyridine, it is a much better electron acceptor, and pyrazine-based complexes of type **B** (X = Cl, -C≡CPh) show intense emissions. Since the non-coordinating N atom in pyrazine can be protonated, alkylated or interact with metal ions and Lewis acids such as B(C_6_F_5_)_3_, the excitation energies are modulated by these interactions, leading to a wide range of emission colours (Figure 1) [39]. The B(C_6_F_5_)_3_ adduct, for example, shows orange emission at 77 K which changes to intense blue on warming and appears to be the first case of a gold(III) complex emitting by a thermally activated delayed fluorescence (TADF) process, where excited singlet and triplet states are of comparable energy, which allows effective triplet harvesting and high quantum yields. Luminescence applications based on gold(III) bidentate and tridentate cyclometallated ligands have developed into a large field which has recently been comprehensively reviewed [44–45].

### Ligand effects and the route to gold hydrides

Given the ease of recording ^1^H NMR data and the absence of steric effects on the Au–H bond, the hydride ligand is an ideal system for assessing the influence of a *trans*-ligand. Likewise, ^1^H NMR chemical shifts are amenable to high-level computations, which provide evidence for the molecular orbital changes that underly the observed chemical shifts.

Kaupp and co-workers employed quasi-relativistic computations on hydride complexes of heavy metals with 5d^10^ and 5d^8^ electron configuration to show that the ^1^H NMR chemical shift is dominated by relativistic spin–orbit (SO) effects. Weak *trans*-influence ligands, such as NO_3_^−^, Cl^−^ or pyridine are strongly shielding, leading to negative values on the ^1^H δ scale, while strong σ-donors like the CH_3_^−^ or BH_2_^−^ anions are strongly deshielding and have the opposite effect (“heavy atom effect on the light-atom shielding”, HALA). For a series of hypothetical complexes [LAuH]^q^ this means the ^1^H NMR chemical shifts cover the range from +10 ppm for NO_3_^−^ or Cl^−^ to −15 ppm for BH_2_^−^ [46]. Whereas in organic compounds ^1^H NMR chemical shifts tend to be interpreted in terms of the acidic or hydridic character of the H atom concerned, the shifts in metal hydrides permit no such interpretations and are the result of spin–orbit and paramagnetic contributions of the metal-centred molecular orbitals. The SO contribution is largest for “naked” AuH, while complexes of typical phosphine donors fall about half-way within these extremes. The calculation can be calibrated against the synthesized Au(I) hydride (NHC)AuH [47], which shows chemical shifts of +5.11 ppm (C_6_D_6_) or +3.38 ppm (CD_2_Cl_2_).

The donor ligands also influence the Au–H bond length, and there is a linear dependence between the calculated Au–H distance and the ^1^H NMR chemical shift, so that NMR data can be used as an experimental estimate of Au–H bonding. Since strong σ-donors destabilise the occupied σ-levels, the character of the gold-centred frontier orbitals changes from d_π_ (shielding) for weak *trans*-ligands to σ (deshielding) for strong donors. This leads to a labilisation (lengthening) of the Au–H bond [46]. A similar effect was found in platinum(II) complexes, where it could be shown that electron donation from L via the metal into the σ* orbital of the *trans*-M–X bond labilises X, an effect that amounts to a repulsion between the metal centre and the ligand or leaving group X in *trans*-position [48].

**Scheme 5:**
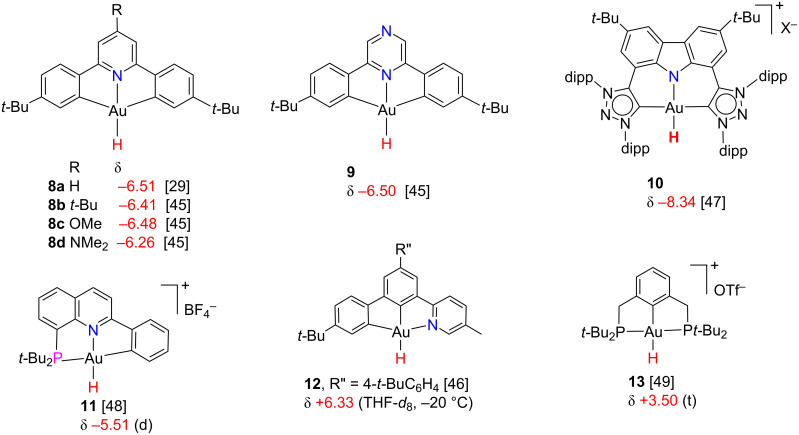
Gold hydride complexes supported by tridentate pincer ligands and corresponding^1^H NMR chemical shifts.

Whereas gold hydrides have remained elusive for decades, in recent years a range of gold(III) hydrides with different C, N and P ligands have been synthesised and spectroscopically characterised, so that the *trans*-influence of various ligand types could be explored. In most cases these require stabilisation by tridentate pincer ligands, illustrated in Scheme 5 alongside their ^1^H NMR chemical shifts [32,49–52]. Both neutral and cationic gold(III) hydride pincer complexes could be prepared. They fall into two categories: those of types **8**–**11** where the hydride ligand is *trans* to an N donor (shielding, negative δ shifts), and those where the hydride is *trans* to an anionic carbon donor (strong electron donation, deshielding, positive δ values), as in types **12** and **13**.

The ligand influence in these pincer hydrides is very evident in their thermal stability. Whereas C^N^C complexes of type **8** and **9** (H *trans* to N) are thermally very stable and can be heated to >80 °C, the analogous C^C^N complex **12** (H *trans* to C) is stable in THF solution at −20 °C but decomposes at room temperature. This instability of **12** may in part also be due to the tendency of the pyridine arm to dissociate; for example, on addition of LiHBEt_3_ the pyridine is displaced to give the dihydrido complex Li[(N–C^C)AuH_2_] [50]. Goldberg’s complex **13**, on the other hand, which is stabilised by two strongly donating P(*t*-Bu)_2_ moieties, is stable at room temperature and could be crystallographically characterised [53]. A gold hydride of type **12** has been postulated as an intermediate in the thermal decomposition of the corresponding Au formate and its reaction with DMAD (DMAD = dimethylacetylene dicarboxylate) [54].

**Scheme 6:**
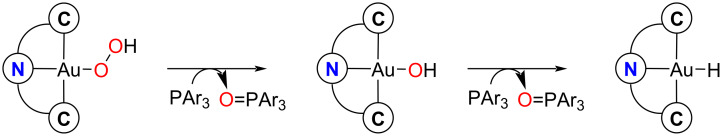
Gold(III) hydride formation by oxygen transfer.

The Au–H bonds in neutral Au(III) hydrides is highly covalent. The stability afforded the gold-bound hydride by the weak *trans*-influence of N in **8** is amply documented and has consequences for its chemical reactivity. For example, **8a** proved stable to air, water and acetic acid, although stronger acids like trifluoroacetic lead to decomposition due to protolytic cleavage of one of the Au–phenyl bonds [31–32]. The stabilising effect of the N-donor in C^N^C ligands is seen quite generally and leads to a change in the usual order of metal–X bond energies. In particular, the bond-dissociation energy of Au–H is higher than that of Au–OH, whereas in comparable platinum complexes [(C^N^N)PtX]^+^ (X = H, OH) the inverse is true. This is the reason why gold(III) peroxides and hydroxides can be converted into gold hydrides by stepwise O-transfer reactions to phosphine (Scheme 6) [55].

**Scheme 7:**
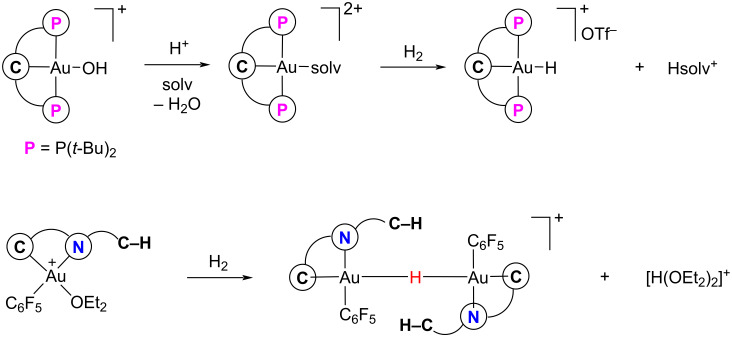
Heterolytic H–H bond cleavage by cationic Au(III) complexes [32,56].

Quite a different mechanism for the conversion of Au–OH into Au–H was found by Goldberg and co-workers for the hydrogenolysis of [(P^C^P)AuOH]^+^, which requires an acid catalyst [56]. A mechanistically similar heterolytic H–H bond cleavage had been reported earlier by Rocchigiani et al. for the highly electrophilic ether adduct [(C^N–CH)Au(C_6_F_5_)OEt_2_]^+^ which shows “frustrated Lewis pair” (FLP)-like reactivity (Scheme 7) [32].

**Scheme 8:**
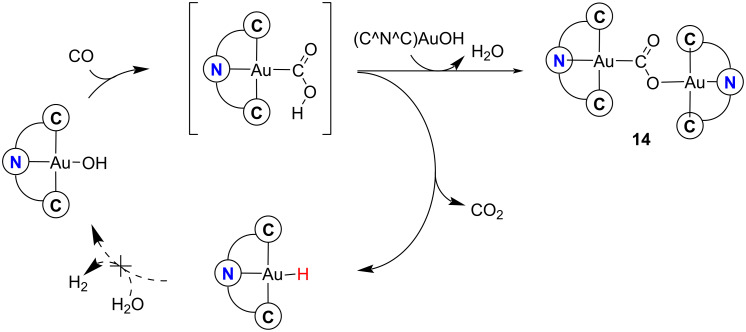
Gold(III) models of the water-gas shift reaction [30].

The electrophilic character of CO ligated to gold explains why gold is more active than platinum in heterogeneous water–gas shift catalysts and is able to work at lower temperatures [57–58]. The water-gas shift could be mimicked by the reaction of (C^N^C)AuOH with CO, which gives CO_2_ together with the gold hydride (C^N^C)AuH. However, closing the cycle by reacting the hydride with water proved not possible due to the *trans*-influence of the N donor, which renders (C^N^C)AuH stable to hydrolysis (Scheme 8) [30]. A by-product of this cycle is the gold(III)–CO_2_ complex **14**, also obtainable from [(C^N^C)Au]_2_O by insertion of CO into the Au–O bond. More recently, Nevado et al. isolated a similar CO_2_ complex based on a C^C^N pincer ligand by the reaction of (C^C^N)AuOSiMe_3_ with CO [59]. In 2021, Hashmi proposed the involvement of the water-gas shift reaction to explain the formation of biphenyl from Au(III) biphenylyl complexes under CO in the presence of traces of water, which explains why CO insertion into the Au–aryl bond was not observed [60].

### Bidentate Au(III) hydride complexes

**Scheme 9:**
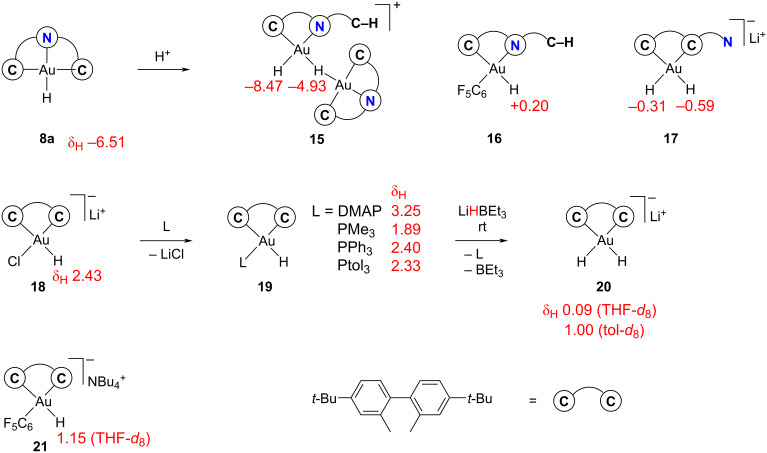
Gold(III) hydride complexes supported by C^C and C^N chelate ligands [32,50].

The selective protolytic cleavage of an Au–C bond in C^N^C complexes with a strong acid [61], or the displacement of pyridine in C^C^N hydrides by H^−^, leads to hydride complexes supported by bidentate C^N or C^C chelate ligands, of type **15**–**17**. Further C^C-bonded gold(III) hydrides could be prepared from 2,2'-biphenylyl complexes, to give compounds of types **18**–**21**, including the first examples of gold(III) hydrido phosphine complexes **19**; the PMe_3_ complex was crystallographically characterised (Scheme 9) [32,50]. Most species were obtained in high purity in solution and characterised spectroscopically. Even the gold dihydrides and the aryl hydrides are remarkably stable and resistant to H–H or H–C(aryl) reductive elimination, even though in Au(III) complexes of monodentate ligands the reductive H–H and H–C elimination barriers were calculated to be quite low [62]. The stability of species like **16**–**21** towards reductive elimination is evidently the result of the steric rigidity imposed by the chelating ligand framework.

With this number of diverse hydride complexes in hand, it was possible to re-examine the dependence of the ^1^H NMR chemical shift as a function of ligand *trans*-influence and extend the theoretical work on gold(I) hydrides [46] to gold(III).

Gratifyingly, the same rules were found to apply. The calculated and experimental ^1^H NMR values were in excellent agreement. Plotting the experimental chemical shift values against the calculated Au–H distances shows the same linear dependence obtained before and, importantly, both Au(I) and Au(III) hydrides fit on the same trend line [50]. Also, since the Au–H bond distances correlate with bond-delocalisation indices, there is therefore also a linear correlation between the ^1^H NMR shift and the covalency of the Au–H bond: The more ionic the Au–H bond, the more positive the hydride shift.

As shown for the Au(I) system, in compounds like (C^C^N)AuH, with H *trans* to anionic C, the frontier orbital has σ-character and is energetically high above the “shielding” Au(d_π_)-type MOs, such that they do not mix, resulting in the high-frequency hydride shift. By contrast, in (C^N^C)AuH the highest σ(Au–H)-type MO is energetically close to the Au(d_π_) orbitals, the Au(d_π_) orbitals are coupled with σ*(Au–H) virtual MOs, and the deshielding effect of the σ-MOs is reduced by SO mixing with the Au(d_π_)-based MOs.

Further computational exploration of the *trans*-influence of various ligands L was conducted on three series of model complexes: *trans*-[HAu(C_6_H_5_)_2_L]^q^, *cis*-[HAu(C^C)L], and [HAu(C^N)L]^q+1^. Ligands L in *trans* position have by far the largest influence, spanning ^1^H chemical shifts of about 22.5 ppm, from the weakest donor H_2_O to the donor with the highest deshielding contribution, SiH_3_^−^. The SO contributions to the ^1^H chemical shift turn from negative to positive in the series H_2_O < NO_3_^−^ ≈ F^−^ < –NCS^−^ ≈ py ≈ NH_3_ < Cl^−^ ≈ H_2_S ≈ I^−^< –SCN^−^ < CN^−^ ≈ CO < C_6_F_5_^−^ ≈ PH_3_ < C_6_H_5_^−^ < CH_3_^−^ < H^−^ < SiH_3_^−^.

Ligands in *cis* position exert a weaker influence, spanning about 6 ppm but with a trend in the opposite direction, with the weakest σ-donors, as well as the strongest π-acceptors, exhibiting the highest deshielding, resulting in the most positive ^1^H hydride shifts. Stronger *trans*-influence ligands in the *cis* position reduce the energy gap between the highest-lying σ(Au–H) MO and the Au(d_π_)-based MO, which is the opposite of what is observed when the same ligands are placed in *trans* position. There is also an influence of the *trans*-ligand on the ligand order of increasing *cis* influence, but generally the above series applies [50].

### Au–H insertion reactions

**Scheme 10:**
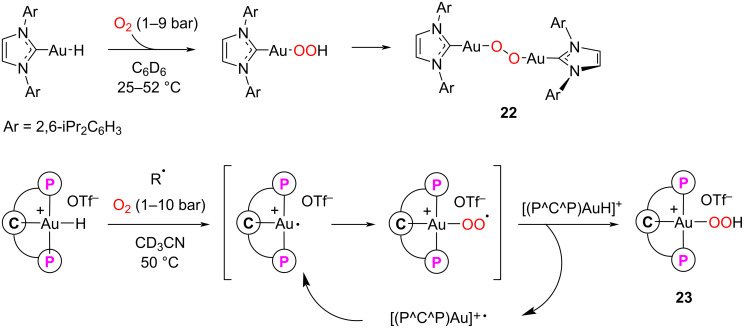
O_2_ insertions into Au–H bonds.

The different nature of the Au–H bond as a function of the *trans*-ligand has consequences in terms of chemical reactivity. Experiments have shown that gold hydrides can be grouped into two classes: those that insert O_2_ into Au–H and those that do not. Roşca et al. showed that O_2_ was able to insert into the Au–H bond of (NHC)Au–H to eventually give the crystallographically characterised peroxide **22** (Scheme 10) [63]. More recently, Goldberg et al. reported a similar O_2_ insertion for [(P^C^P)AuH]^+^ by an auto-accelerating radical chain mechanism, to give a stable hydroperoxide complex **23** that was characterised by X-ray diffraction. This reaction was greatly accelerated by AIBN as radical initiator which generates Au(II) radical cations [(P^C^P)Au]^+•^, as well as RO^•^ and ROO^•^ radicals, which propagate the reaction. The final product is then formed in a bimolecular reaction: LAuOO^•^ + HAuL → LAuOOH + LAu^•^ [53].

In both these cases the Au–H bond is activated by the presence of a strong C-donor in *trans* position. So far no O_2_ insertion has been reported for Au–H *trans* to N.

Alkene and alkyne insertions into M–H or M–C are a mainstay of homogeneous catalysis. Alkyne insertion by a migratory insertion process leads to *cis*-addition. By contrast, insertions of alkyne into Au–H bonds result in *anti* (*trans*)-addition products, which cannot readily be explained by the conventional migratory pathway.

To the extent that this has been tested experimentally, those that insert O_2_ also insert activated alkynes such as DMAD. For example, Sadighi reported that (NHC)AuH reacted with DMAD to give the *anti*-insertion product stereoselectively but failed to react with 3-hexyne and diphenylacetylene [47]. The recently reported NHC-decorated diphosphene complex (NHC)P(R)–P(R)AuH similarly forms the *Z*-vinyl product with DMAD (R = 2,6-Mes_2_C_6_H_3_) [64]. Consistent with this, while (C^N^C)AuH inserts neither O_2_ nor DMAD (nor indeed ethylene, CO_2_ or benzaldehyde) [31,49], heating (C^C^N)Au–O_2_CH with RC≡CR (R = *t-*BuOOC–) gives (C^C^N)Au–C(R)=C(R)H (*Z*-configuration), most probably via (C^C^N)AuH as intermediate [54].

Allenes are more reactive, and both (C^N^C)AuH **8a** and [(P^N^C)AuH]^+^ **11** give allene-insertion products, by unspecified mechanisms [31,52].

**Scheme 11:**
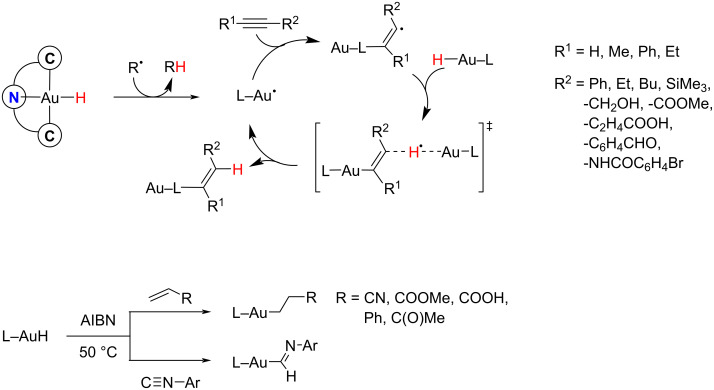
Alkyne, alkene and isocyanide hydroauration by a bimolecular gold radical mechanism [49,65–66].

This picture changed however when it was realised that (C^N^C)AuH complexes of type **8** can react with acetylenes slowly, in a possibly light-induced radical reaction [49]. The addition of radical initiators such as azobis(isobutyronitrile) (AIBN) reduced the reaction times from days to minutes, with quantitative and completely stereoselective formation of *Z*-vinyl gold complexes. Both terminal and internal alkynes could be inserted, with a wide variety of functional groups including alkyl, aryl, OH, COOH, amide and aldehyde substituents, but not DMAD. The excellent stereo- and regiochemical selectivity could be explained by a bimolecular mechanism, as confirmed by DFT calculations. Using the same radical-initiated approach it was also possible to realise gold hydride insertions of alkenes [65] and of isocyanides (Scheme 11) [66].

**Scheme 12:**
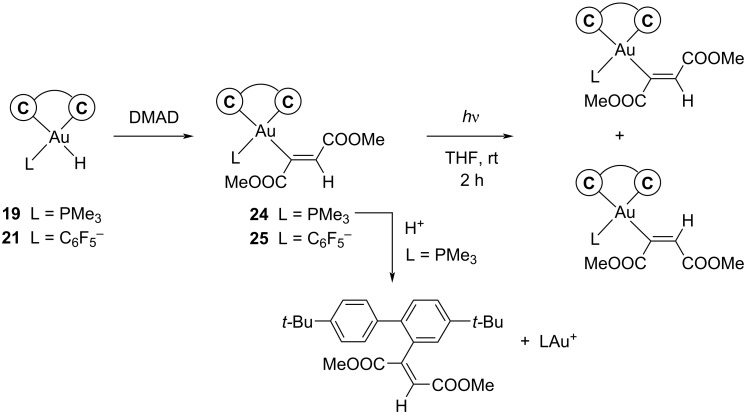
Reactions of (C^C)Au–H with DMAD [50].

A different reactivity was observed for C^C-stabilised hydrides, with H *trans* to anionic C. The complexes (C^C)AuH(L) **19** (L = PMe_3_, Ptol_3_) and [(C^C)AuH(C_6_F_5_)]^−^ **21** react slowly at room temperature with DMAD to give the corresponding *Z*-vinyl products **24** and **25**, respectively. On treatment with acid **24** decomposes with reductive C–C coupling but without Au–vinyl protolysis, while irradiation of THF solutions of **24** or **25** at room temperature leads to C=C-bond isomerisation, to give the gold vinyls in *E*/*Z* ratios of 20:80 (**24**) and 50:50 (**25**), respectively (Scheme 12). The *Z*-isomer is evidently the kinetic product.

The gold(III) hydrides that react with DMAD are all neutral or negatively charged complexes carrying electron-rich anionic C-ligands and/or phosphines. Given that DMAD has a low reduction potential (−0.8 V) compared to that of diphenylacetylene (−2.1 V), it has been proposed that these DMAD insertions involve electron transfer and generation of the DMAD radical anion in the solvent cage, followed by ligand recombination [50].

Although not involving Au–H bonds, it is noted in passing that alkyne *cis*-insertions into Au(I)–Si and Au(I)–B bonds have been reported, as in the reactions of (IPr)AuSiMe_2_Ph with HC≡CCOOMe [67], and of (IPr)Au–boryl with internal alkynes, together with the subsequent isomerisation to *trans*-vinyls [68].

**Scheme 13:**
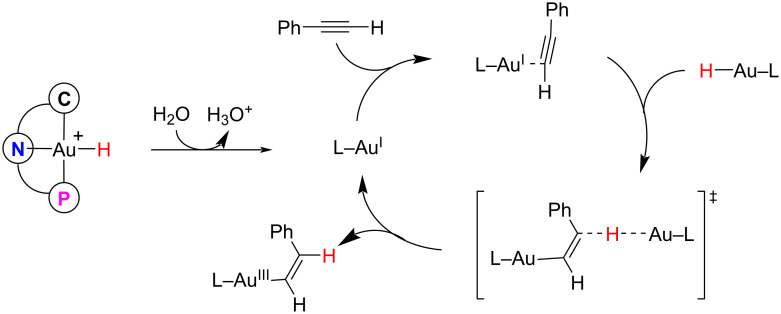
Alkyne hydroauration by a bimolecular Au(III)–Au(I)-assisted process [69].

However, there is apparently yet more versatility in the mechanisms of alkyne hydroauration and may operate for the [(P^N^C)AuH]^+^ cation **11**. Nevado et al. found recently that **11** inserts both DMAD as well as terminal alkynes, to give Au(III) *Z*-vinyl complexes. Control experiments ruled out the involvement of radicals. Also, the reaction was surprisingly accelerated by water. The findings are in agreement with another type of cooperative bimolecular mechanism, where the vacant coordination site required for alkyne binding is generated not by a radical initiator but apparently by the reductive deprotonation [29] of [(P^N^C)AuH]^+^ by water, to give a T-shaped Au(I) intermediate, (P^N^C)Au, capable of sequestering the alkyne. This in turn is attacked by another Au(III) hydride to complete the *anti*-addition process (Scheme 13) [69].

### Ligand effects of C^N, C^P and P^N chelates

Gold(III) complexes of C^N cyclometallated ligands (type **D**, Scheme 2) represent a large compound class that has been investigated for many years [70]. The key feature of C^N ligands is that these chelates combine a strong anionic C-donor with a weak pyridine donor in a rigid framework. In a complex (C^N)AuX_2_ the two X ligands therefore display very different reactivity. Cyclometallated phosphines (C^P) (type **G**), on the other hand, consist of donors of about equal strengths, particularly if P is a dialkylphosphine substituent. They are represented here by the 9-phosphinonaphthyl ligand introduced by Bourissou [71]; the chemistry of transition-metal C^P complexes has recently been reviewed [72]. P^N Ligands, notably 2-Me_2_NC_6_H_4_P(1-adamantyl)_2_ (“MeDalPhos”) is one of a group of chelating ligands that greatly facilitate oxidative addition reactions to gold(I) phosphine complexes by stabilising the Au(III) state [73–78]. Since these complexes are a means of facilitating oxidative addition reactions to gold by chelate formation, they have become a mainstay in Au(I,III) redox catalysis. The use of P^N and related ligands in synthesis has recently been reviewed [79].

**Scheme 14:**
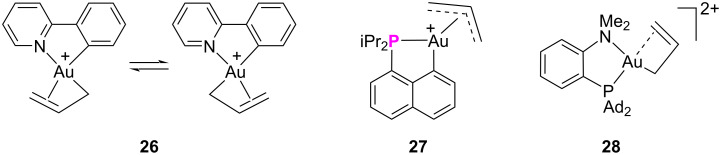
Gold(III) π-allyl complexes.

A good way of assessing the *trans*-influence in these ligands is to consider the structure of the recently described π-allyl complexes **26**–**28** (Scheme 14) [80–83]. Compounds **26** and **27** were characterised by X-ray diffraction and NMR spectroscopy, which showed that **26** is fluxional. The dication **28** was spectroscopically identified and the structure optimised by DFT calculations [82]. They clearly fall into two categories: In the C^N and P^N compounds the allyl bonding is very asymmetrical, with a long and a short C–C bond and strong variations in Au–C bond distances, as expected for a σ,π-allyl, while in **27** and its methallyl analogue the bonding is that of a typical η^3^ structure.

These differences in bonding between C^N and C^P chelates translates into significant differences in reactivity.

**Scheme 15:**
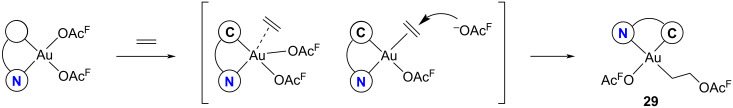
Outer-sphere mechanism of ethylene insertion into Au–O bonds [20,84].

The gold–O bond in trifluoroacetate complexes is labile and allows the formal “insertion” of ethylene to give Au-C_2_H_4_OAc^F^ products [28]. In a careful mechanistic study on the reaction of ethylene with (C^N)Au(OAc^F^)_2_ Tilset et al. pointed out that due to the *trans*-influence the reactivity of the two Ac^F^O ligands differs: the one *trans* to C is kinetically labile and exchanges with free Ac^F^O^−^, whereas the one *trans* to N does not. On the other hand, there is a thermodynamic preference for placing stronger donors like alkyl and ethylene in *trans*-position to N as the weaker donor. The reaction with ethylene is greatly accelerated if Ac^F^OH is used as polar solvent. The product is the monoalkyl **29**, with the alkyl *trans* to N. Dialkyl complexes (C^N)Au(C_2_H_4_OAc^F^)_2_ were not detected. Only one of the Ac^F^O ligands undergoes insertion. The observed product could be formed either by an intramolecular migratory pathway, or by an outer-sphere nucleophilic attack by Ac^F^O^−^ on a coordinated ethylene ligand. DFT studies eliminated the intramolecular migratory pathway as uncompetitive and pointed to an associative interchange mechanism to bring the ethylene into the position *trans* to N (Scheme 15). The external nucleophilic attack pathway was proved experimentally using *cis*-1,2-C_2_H_2_D_2_, which generates the *threo* product, consistent with *anti*-addition of an external nucleophile [84]. The same principles apply to other alkenes and nucleophiles (alcohols, water) [85].

**Scheme 16:**
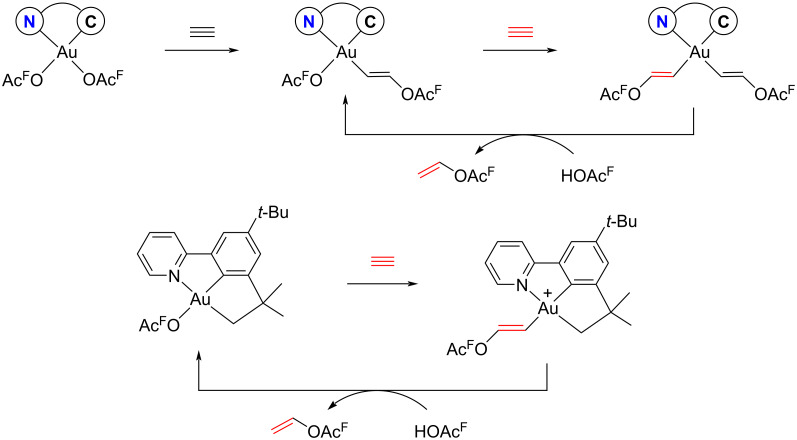
Catalytic acetylene functionalisation [86–87].

Using acetylene instead of ethylene gives first the analogous mono-insertion product, with the vinyl ligand *trans* to N. The mono-vinyl complex (C *trans* to N) could be isolated. Adding excess acetylene led to the catalytic formation of vinyl trifluoroacetate, with catalysis happening exclusively at the more labile site *trans* to C(phenyl) of the C^N chelate [86]. The same reaction is also catalysed by a related pincer complex, (N^C^C)Au(OAc^F^) (Scheme 16). This tridentate structure, and the stabilisation of the Au–C(sp^3^) bond by the weak *trans*-influence of N, renders the catalyst more stable against Au-aryl protonolysis and thus catalyst deactivation, resulting in higher turnover numbers [87].

The inequivalence of the two *trans*-coordination sites in C^N complexes contrasts starkly with the behaviour of the C^P system. Here the metal centre is subject to the effect of two good donors of about equal strength. This is the only system reported so far where gold(III) displays palladium-like reactivity, such as alkene insertions into Au–alkyl bonds and β-H elimination.

**Scheme 17:**
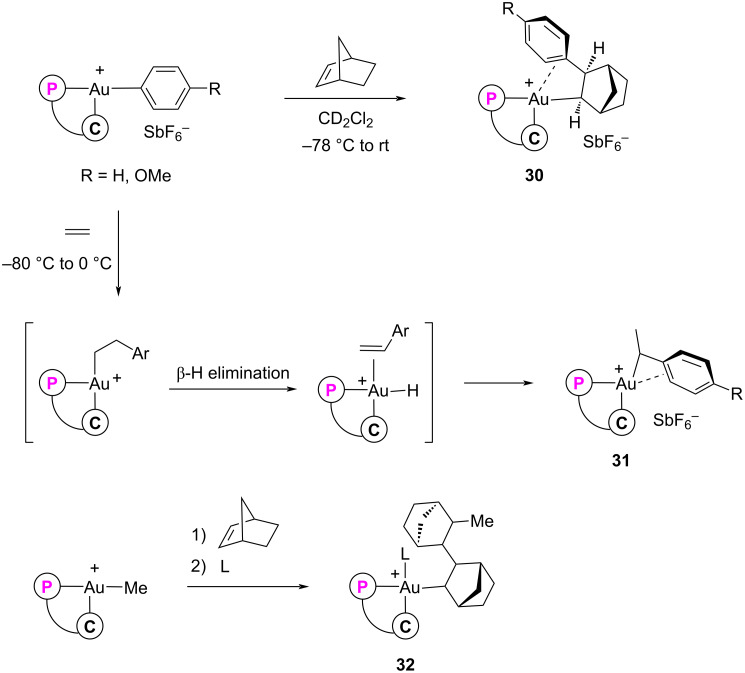
Examples of alkene insertions into Au–C bonds [88–89].

**Scheme 18:**
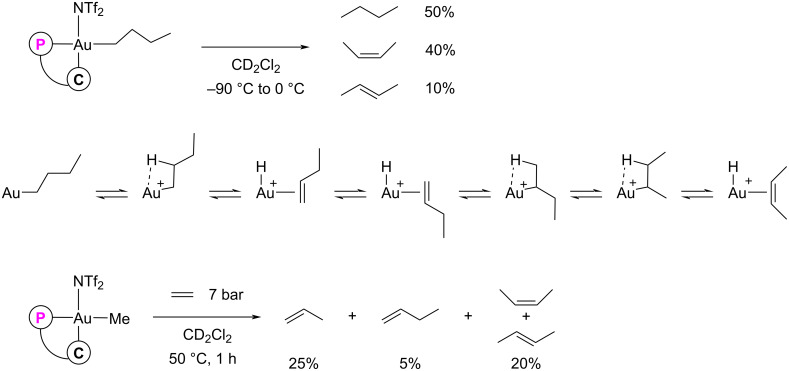
Examples of β-H elimination and chain walking processes in (C^P)-ligated gold alkyls [90].

The influence of C^(−)^ and/or -PiPr_2_ donors weakens Au–C bonds in *trans* position to a sufficient extent to make alkene insertion reactions energetically competitive. This is exemplified by the alkene insertions into an Au–aryl bond to give **30** and **31** (Scheme 17) [88], and by the double norbornene insertion into Au–Me to give **32** [89]. Further, the decomposition products of [(C^P)Au*n-*Bu]^+^ and the reaction of [(C^P)AuMe]NTf_2_ with ethylene under pressure demonstrate alkene insertion/β-H elimination and chain walking sequences (Scheme 18) [90]. These reactions contrast with those of the benzoquinolinato complex [(C^N)Au*n-*Bu]SbF_6_, which was thermally stable and proved resistant to β-H elimination or H/D exchange [91].

**Scheme 19:**
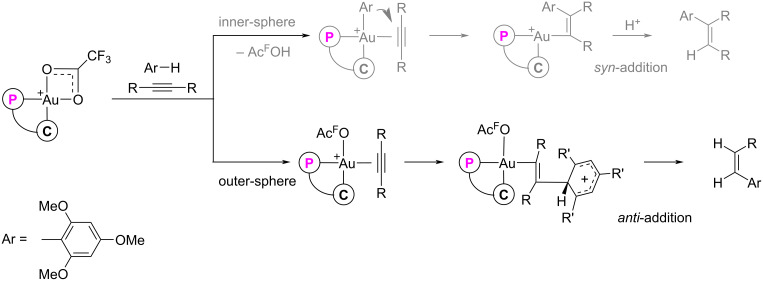
Mechanism of alkyne hydroarylation with (C^P) gold catalysts [92].

**Scheme 20:**
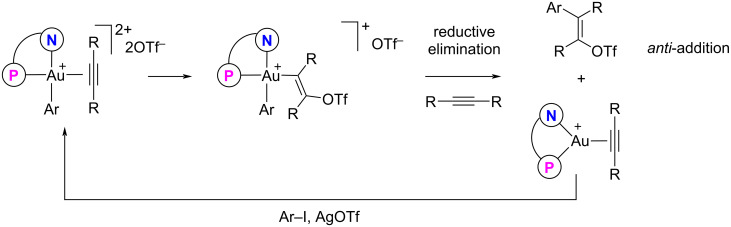
Vinylic triflate esters by gold-catalysed nucleophilic attack on alkynes [94].

The same system also catalyses the hydroarylation of alkynes with 1,3,5-trimethoxybenzene (TMB), by *anti*-addition of a C–H bond of TMB to give the *Z*-vinyl. Given the precedence for insertion chemistry with the C^P ligand, the alkyne hydroarylation might have been expected to follow a similar migratory route: formation of a gold–aryl, then alkyne insertion in the Au–Ar bond by an inner-sphere pathway. Alternatively, the process could involve alkyne coordination, then outer-sphere attack by TMB, followed protodeauration. Modelling various scenarios by DFT showed a clear energetic preference for the outer-sphere mechanism, with the arene acting like a nucleophile (Scheme 19) [92].

(P^N)Au complexes display quite similar reactivity with alkynes, leading to 1,2-heteroarylation products, as recently shown in the catalytic alkyne arylations with alcohols as nucleophiles to give vinyl ethers [78]. Given that alkynes coordinated to cationic Au(III) centres display strong vinyl cation character [28], it is perhaps not unexpected that even weak donors like the triflate anion undergo nucleophilic attack, as recently demonstrated by Rocchigiani et al. for the catalytic formation of *trans*-vinyl triflate esters (Scheme 20) [93].

The above discussion has highlighted how important the ligand *trans*-influence is in determining gold-catalysed reaction paths. Nickel or palladium-like reactivity, such as migratory alkene, alkyne or CO insertions into metal–carbon bonds, β-H eliminations or chain walking, are very much the exception and depend on strong donor ligands. In spite of its versatility in gold catalysis, the much-used MeDalPhos P^N ligand would not appear to fall into that category.

Nevertheless, unusual, palladium-like reactivity has recently been claimed to explain the (MeDalPhos)Au-catalysed synthesis of Heck-type olefins, including migratory alkene 2,1-insertion into a Au–aryl bond, followed by β-H elimination, to give non-conjugated benzyl alkenes [94]. This reactivity scenario has been extended to include chain walking [95]. The main evidence for the migratory alkene insertion proposal was a DFT model; however, since such models rarely explore the whole energy landscape, it cannot be a substitute for experimental evidence, and any claims of dramatic new reactivity must be treated with caution.

In the (P^N)Au(I,III) catalytic system, β-elimination and chain walking make it necessary to invoke a hypothetical dicationic hydride intermediate, [(P^N)AuH(alkene)]^2+^. Based on the chemistry of the gold(III) hydrides described above, none of which showed any tendency for migratory alkene insertion [14], and in view of the resistance even of coordinatively unsaturated alkyls like [(C^N)Au*n-*Bu]^+^ to β-H elimination [91], such proposals needed to be regarded with scepticism. This was supported by the authors’ own calculations which showed that [(P^N)AuH(alkene)]^2+^ is a high-energy species which undergoes a near-barrierless rearrangement to a stable but catalytically inactive N-protonated species: [(P^N)AuH(alkene)]^2+^ → [(P–NH^(+)^Au]^+^ + alkene (Δ*G* = −25.4 kcal/mol) [94]. The formation of [(P–NH^(+)^Au]^+^ would therefore shut down any catalysis. Another factor is that the Pd-catalysed Heck reaction requires alkene 2,1-insertion [96], whereas calculations on gold alkene insertions invariably show 1,2-insertions to be energetically more favourable. However, these do not generate Heck-type arylated olefins.

**Scheme 21:**
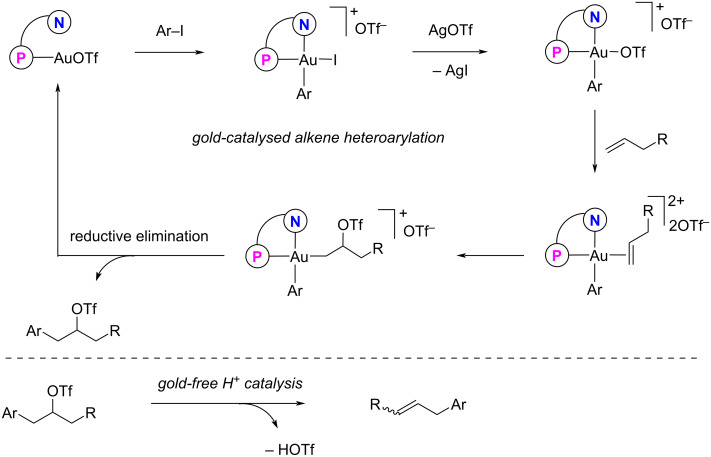
Gold-catalysed and gold-free steps in the formation of Heck-type olefins [97,99].

A detailed spectroscopic, kinetic and computational study by Budzelaar et al. has recently clarified that these reactions in fact follow a nucleophile addition pathway [97]. Attack on the coordinated olefin by a triflate anion, followed by aryl–alkyl reductive elimination, generates an alkyl triflate as the primary organic product. Kinetic measurements showed that this occurs before the formation of any olefinic end-products. In a second, separate H^+^-catalysed step HOTf is eliminated, to give the observed non-conjugated alkene. This second step also proceeds in the absence of a gold catalyst, with identical outcomes (Scheme 21). Given the strong Lewis acidity of cationic Au(III) centres, in the presence of traces of moisture H^+^-initiated reactions are always likely. Nucleophilic attack on a cationic Au(III)–alkene complex is well known [27] and is essentially barrierless and irreversible, so that a migratory insertion pathway becomes uncompetitive. These are of course the basis of gold-catalysed alkene heteroarylations, a well-established reaction sequence [98].

A wider ranging computational study involving various alkene substrates and ligand variations confirmed the nucleophilic attack on alkenes by triflate as the energetically preferred pathway for P^N complexes [99]. These results also showed that for the more donating P^P chelates a migratory alkene 1,2-insertion pathway (but not 2,1-insertions to give branched Au–alkyls) could potentially become competitive, in line with the results discussed above for electron-rich C^P complexes.

## Conclusion

In summary, the *trans*-influence is a crucial factor in gold chemistry, particularly in gold(III) and gold(I,III) redox systems. The *trans*-influence controls ligand bond energies, determines the nature of gold frontier orbitals and how they can interact with ligands; it determines the stability of possible catalytic intermediates and directs reaction pathways while ruling out alternatives, however convenient, desirable or familiar such alternative pathways may appear. This survey confirms the idea that gold is distinctly different from palladium, and while the end-products may resemble those of palladium-catalysed reactions, gold seems to have a surprisingly agile ability to demonstrate mechanistic diversity.

## Data Availability

Data sharing is not applicable as no new data was generated or analyzed in this study.
